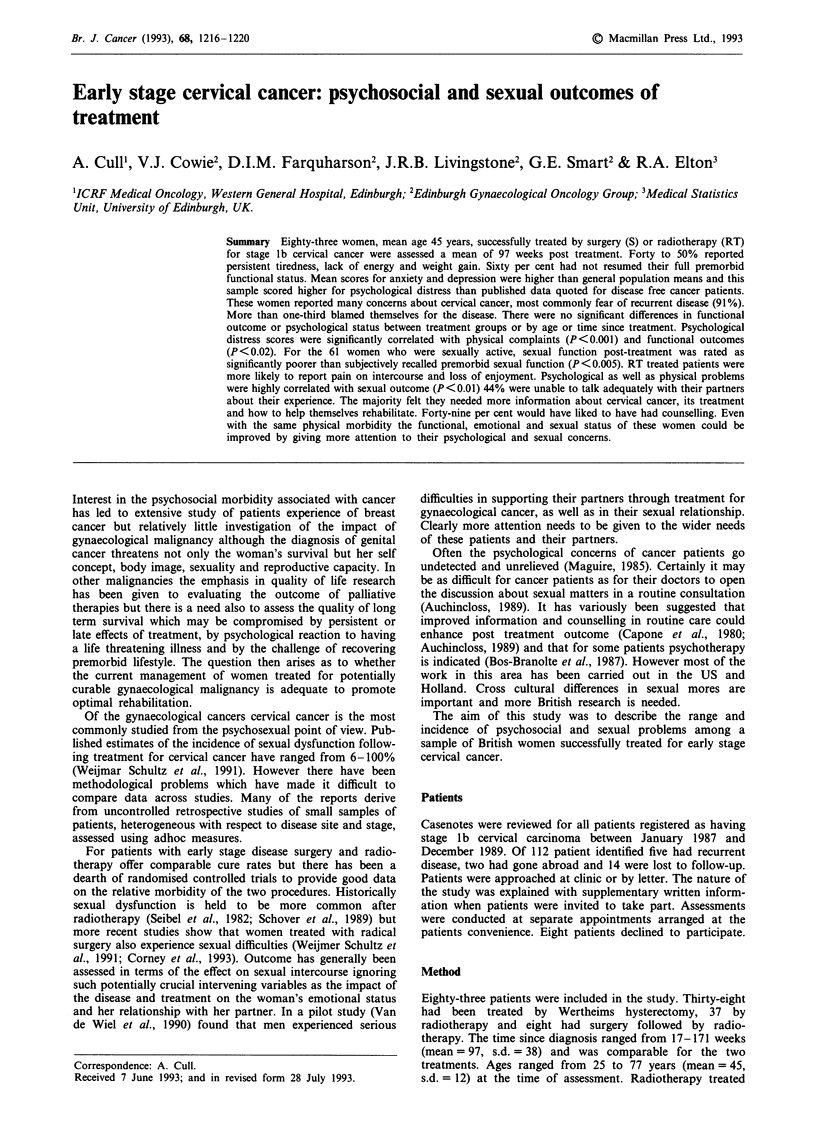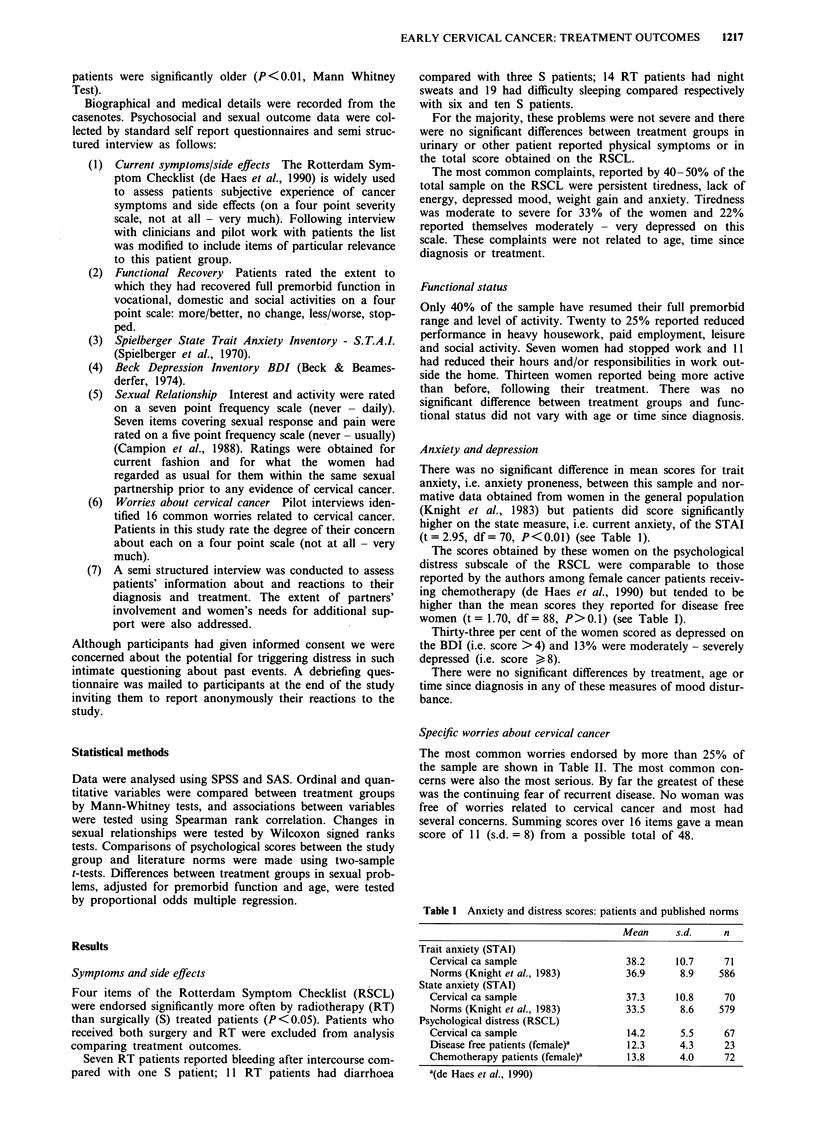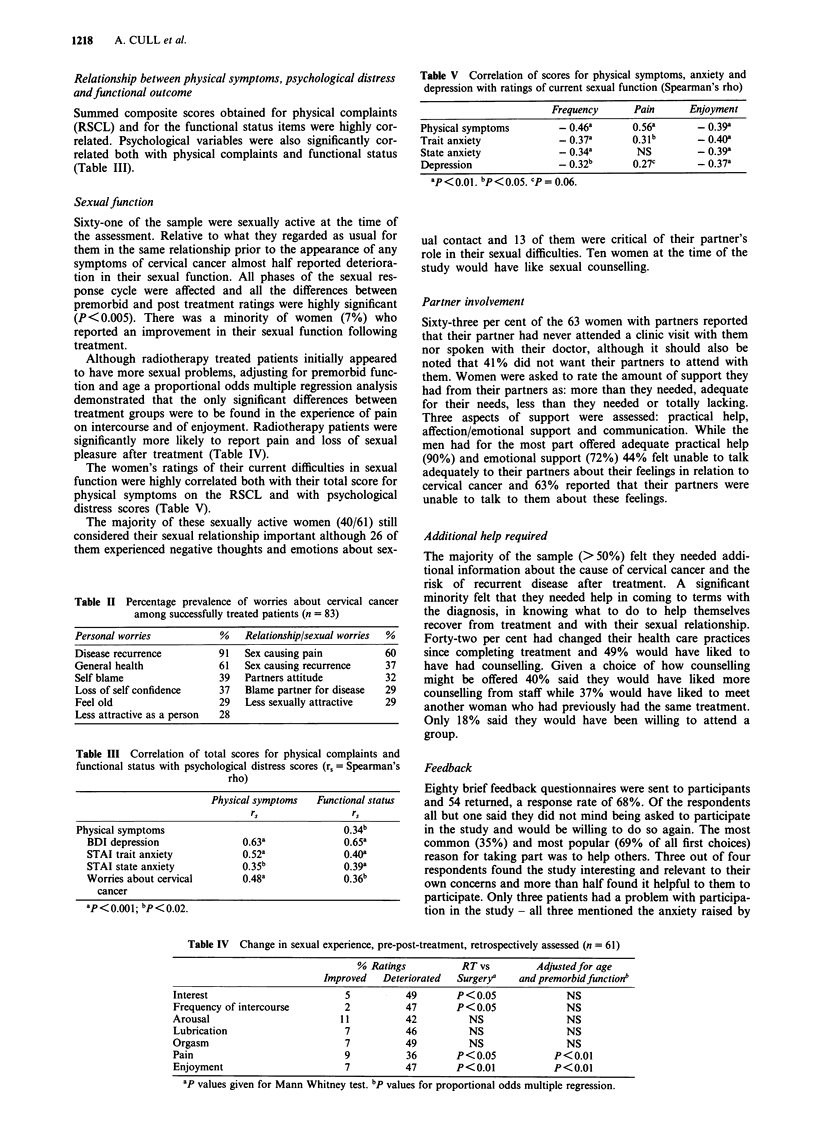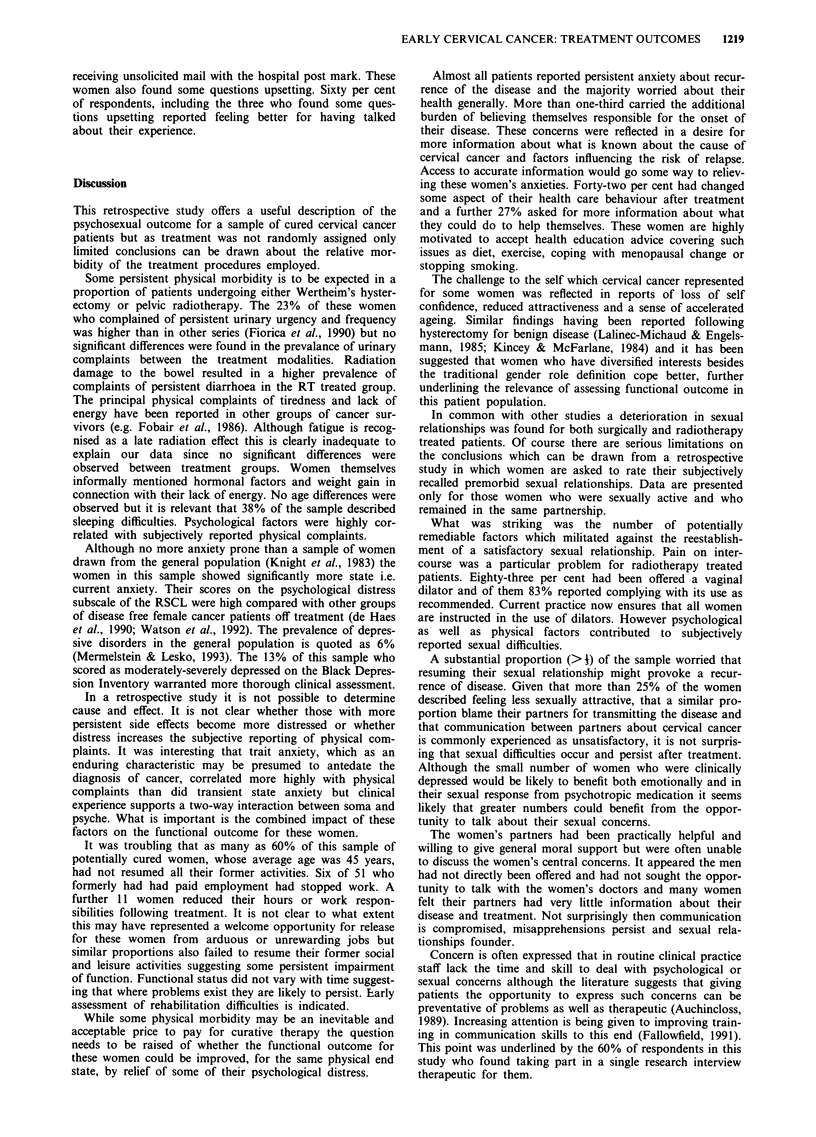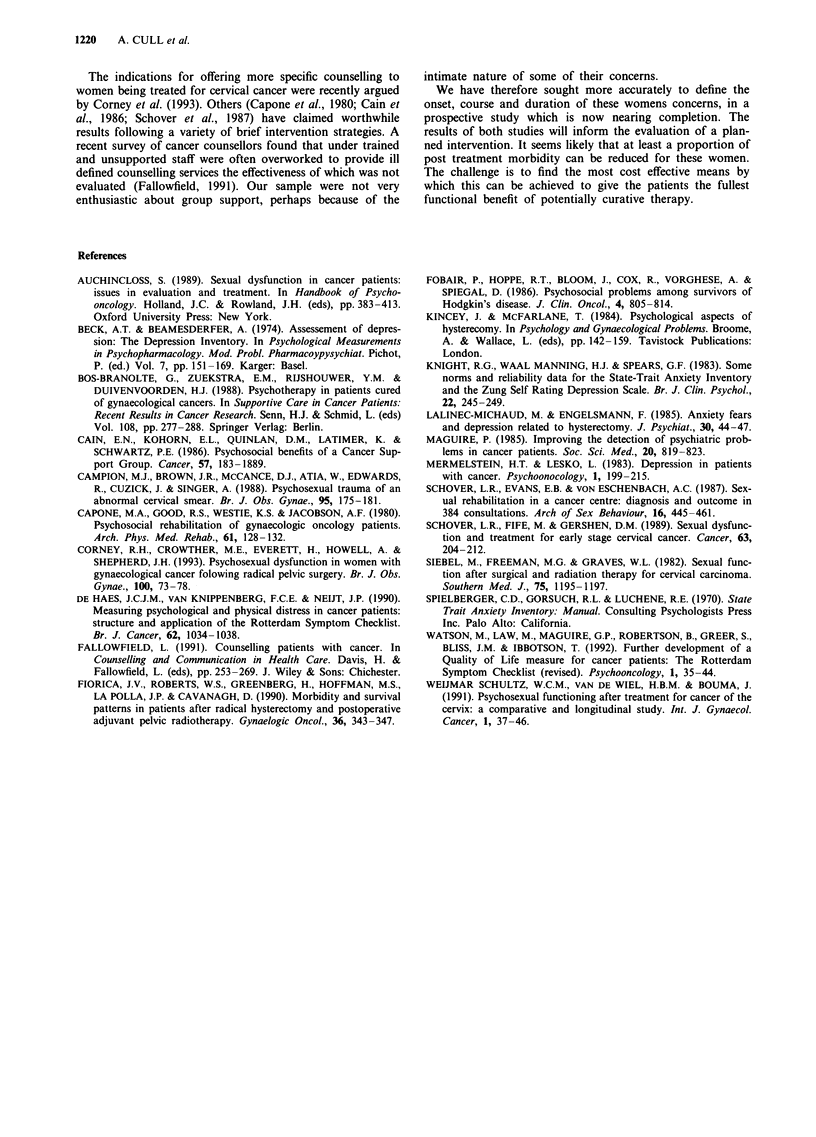# Early stage cervical cancer: psychosocial and sexual outcomes of treatment.

**DOI:** 10.1038/bjc.1993.507

**Published:** 1993-12

**Authors:** A. Cull, V. J. Cowie, D. I. Farquharson, J. R. Livingstone, G. E. Smart, R. A. Elton

**Affiliations:** ICRF Medical Oncology, Western General Hospital, Edinburgh, UK.

## Abstract

Eighty-three women, mean age 45 years, successfully treated by surgery (S) or radiotherapy (RT) for stage 1b cervical cancer were assessed a mean of 97 weeks post treatment. Forty to 50% reported persistent tiredness, lack of energy and weight gain. Sixty per cent had not resumed their full premorbid functional status. Mean scores for anxiety and depression were higher than general population means and this sample scored higher for psychological distress than published data quoted for disease free cancer patients. These women reported many concerns about cervical cancer, most commonly fear of recurrent disease (91%). More than one-third blamed themselves for the disease. There were no significant differences in functional outcome or psychological status between treatment groups or by age or time since treatment. Psychological distress scores were significantly correlated with physical complaints (P < 0.001) and functional outcomes (P < 0.02). For the 61 women who were sexually active, sexual function post-treatment was rated as significantly poorer than subjectively recalled premorbid sexual function (P < 0.005). RT treated patients were more likely to report pain on intercourse and loss of enjoyment. Psychological as well as physical problems were highly correlated with sexual outcome (P < 0.01) 44% were unable to talk adequately with their partners about their experience. The majority felt they needed more information about cervical cancer, its treatment and how to help themselves rehabilitate. Forty-nine per cent would have liked to have had counselling. Even with the same physical morbidity the functional, emotional and sexual status of these women could be improved by giving more attention to their psychological and sexual concerns.


Br. J. Cancer (1993), 68, 1216–1220 © Macmillan Press Ltd., 1993

### Early stage cervical cancer: psychosocial and sexual outcomes of treatment

A. Cull[1], V.J. Cowie[2], D.I.M. Farquharson[2], J.R.B. Livingstone[2], G.E. Smart[2] & R.A. Elton[3]

[1]*ICRF Medical Oncology, Western General Hospital, Edinburgh;* [2]*Edinburgh Gynaecological Oncology Group;* [3]*Medical Statistics Unit, University of Edinburgh, UK.*

**Summary** Eighty-three women, mean age 45 years, successfully treated by surgery (S) or radiotherapy (RT) for stage 1b cervical cancer were assessed a mean of 97 weeks post treatment. Forty to 50% reported persistent tiredness, lack of energy and weight gain. Sixty per cent had not resumed their full premorbid functional status. Mean scores for anxiety and depression were higher than general population means and this sample scored higher for psychological distress than published data quoted for disease free cancer patients. These women reported many concerns about cervical cancer, most commonly fear of recurrent disease (91%). More than one-third blamed themselves for the disease. There were no significant differences in functional outcome or psychological status between treatment groups or by age or time since treatment. Psychological distress scores were significantly correlated with physical complaints ($P<0.001$) and functional outcomes ($P<0.02$). For the 61 women who were sexually active, sexual function post-treatment was rated as significantly poorer than subjectively recalled premorbid sexual function ($P<0.005$). RT treated patients were more likely to report pain on intercourse and loss of enjoyment. Psychological as well as physical problems were highly correlated with sexual outcome ($P<0.01$) 44% were unable to talk adequately with their partners about their experience. The majority felt they needed more information about cervical cancer, its treatment and how to help themselves rehabilitate. Forty-nine per cent would have liked to have had counselling. Even with the same physical morbidity the functional, emotional and sexual status of these women could be improved by giving more attention to their psychological and sexual concerns.

Interest in the psychosocial morbidity associated with cancer has led to extensive study of patients experience of breast cancer but relatively little investigation of the impact of gynaecological malignancy although the diagnosis of genital cancer threatens not only the woman's survival but her self concept, body image, sexuality and reproductive capacity. In other malignancies the emphasis in quality of life research has been given to evaluating the outcome of palliative therapies but there is a need also to assess the quality of long term survival which may be compromised by persistent or late effects of treatment, by psychological reaction to having a life threatening illness and by the challenge of recovering premorbid lifestyle. The question then arises as to whether the current management of women treated for potentially curable gynaecological malignancy is adequate to promote optimal rehabilitation.

Of the gynaecological cancers cervical cancer is the most commonly studied from the psychosexual point of view. Published estimates of the incidence of sexual dysfunction following treatment for cervical cancer have ranged from 6–100% (Weijmar Schultz *et al.*, 1991). However there have been methodological problems which have made it difficult to compare data across studies. Many of the reports derive from uncontrolled retrospective studies of small samples of patients, heterogeneous with respect to disease site and stage, assessed using adhoc measures.

For patients with early stage disease surgery and radiotherapy offer comparable cure rates but there has been a dearth of randomised controlled trials to provide good data on the relative morbidity of the two procedures. Historically sexual dysfunction is held to be more common after radiotherapy (Seibel *et al.*, 1982; Schover *et al.*, 1989) but more recent studies show that women treated with radical surgery also experience sexual difficulties (Weijmer Schultz *et al.*, 1991; Corney *et al.*, 1993). Outcome has generally been assessed in terms of the effect on sexual intercourse ignoring such potentially crucial intervening variables as the impact of the disease and treatment on the woman's emotional status and her relationship with her partner. In a pilot study (Van de Wiel *et al.*, 1990) found that men experienced serious difficulties in supporting their partners through treatment for gynaecological cancer, as well as in their sexual relationship. Clearly more attention needs to be given to the wider needs of these patients and their partners.

Often the psychological concerns of cancer patients go undetected and unrelieved (Maguire, 1985). Certainly it may be as difficult for cancer patients as for their doctors to open the discussion about sexual matters in a routine consultation (Auchincloss, 1989). It has variously been suggested that improved information and counselling in routine care could enhance post treatment outcome (Capone *et al.*, 1980; Auchincloss, 1989) and that for some patients psychotherapy is indicated (Bos-Branolte *et al.*, 1987). However most of the work in this area has been carried out in the US and Holland. Cross cultural differences in sexual mores are important and more British research is needed.

The aim of this study was to describe the range and incidence of psychosocial and sexual problems among a sample of British women successfully treated for early stage cervical cancer.

### Patients

Casenotes were reviewed for all patients registered as having stage 1b cervical carcinoma between January 1987 and December 1989. Of 112 patient identified five had recurrent disease, two had gone abroad and 14 were lost to follow-up. Patients were approached at clinic or by letter. The nature of the study was explained with supplementary written information when patients were invited to take part. Assessments were conducted at separate appointments arranged at the patients convenience. Eight patients declined to participate.

### Method

Eighty-three patients were included in the study. Thirty-eight had been treated by Wertheims hysterectomy, 37 by radiotherapy and eight had surgery followed by radiotherapy. The time since diagnosis ranged from 17–171 weeks (mean = 97, s.d. = 38) and was comparable for the two treatments. Ages ranged from 25 to 77 years (mean = 45, s.d. = 12) at the time of assessment. Radiotherapy treated

Correspondence: A. Cull.
Received 7 June 1993; and in revised form 28 July 1993.

patients were significantly older ($P<0.01$, Mann Whitney Test).

Biographical and medical details were recorded from the casenotes. Psychosocial and sexual outcome data were collected by standard self report questionnaires and semi structured interview as follows:

(1) *Current symptoms/side effects* The Rotterdam Symptom Checklist (de Haes *et al.*, 1990) is widely used to assess patients subjective experience of cancer symptoms and side effects (on a four point severity scale, not at all – very much). Following interview with clinicians and pilot work with patients the list was modified to include items of particular relevance to this patient group.
(2) *Functional Recovery* Patients rated the extent to which they had recovered full premorbid function in vocational, domestic and social activities on a four point scale: more/better, no change, less/worse, stopped.
(3) *Spielberger State Trait Anxiety Inventory - S.T.A.I.* (Spielberger *et al.*, 1970).
(4) *Beck Depression Inventory BDI* (Beck & Beamesderfer, 1974).
(5) *Sexual Relationship* Interest and activity were rated on a seven point frequency scale (never – daily). Seven items covering sexual response and pain were rated on a five point frequency scale (never – usually) (Campion *et al.*, 1988). Ratings were obtained for current fashion and for what the women had regarded as usual for them within the same sexual partnership prior to any evidence of cervical cancer.
(6) *Worries about cervical cancer* Pilot interviews identified 16 common worries related to cervical cancer. Patients in this study rate the degree of their concern about each on a four point scale (not at all – very much).
(7) A semi structured interview was conducted to assess patients' information about and reactions to their diagnosis and treatment. The extent of partners' involvement and women's needs for additional support were also addressed.

Although participants had given informed consent we were concerned about the potential for triggering distress in such intimate questioning about past events. A debriefing questionnaire was mailed to participants at the end of the study inviting them to report anonymously their reactions to the study.

### Statistical methods

Data were analysed using SPSS and SAS. Ordinal and quantitative variables were compared between treatment groups by Mann-Whitney tests, and associations between variables were tested using Spearman rank correlation. Changes in sexual relationships were tested by Wilcoxon signed ranks tests. Comparisons of psychological scores between the study group and literature norms were made using two-sample *t*-tests. Differences between treatment groups in sexual problems, adjusted for premorbid function and age, were tested by proportional odds multiple regression.

### Results

### *Symptoms and side effects*

Four items of the Rotterdam Symptom Checklist (RSCL) were endorsed significantly more often by radiotherapy (RT) than surgically (S) treated patients ($P<0.05$). Patients who received both surgery and RT were excluded from analysis comparing treatment outcomes.

Seven RT patients reported bleeding after intercourse compared with one S patient; 11 RT patients had diarrhoea compared with three S patients; 14 RT patients had night sweats and 19 had difficulty sleeping compared respectively with six and ten S patients.

For the majority, these problems were not severe and there were no significant differences between treatment groups in urinary or other patient reported physical symptoms or in the total score obtained on the RSCL.

The most common complaints, reported by 40–50% of the total sample on the RSCL were persistent tiredness, lack of energy, depressed mood, weight gain and anxiety. Tiredness was moderate to severe for 33% of the women and 22% reported themselves moderately – very depressed on this scale. These complaints were not related to age, time since diagnosis or treatment.

### *Functional status*

Only 40% of the sample have resumed their full premorbid range and level of activity. Twenty to 25% reported reduced performance in heavy housework, paid employment, leisure and social activity. Seven women had stopped work and 11 had reduced their hours and/or responsibilities in work outside the home. Thirteen women reported being more active than before, following their treatment. There was no significant difference between treatment groups and functional status did not vary with age or time since diagnosis.

### *Anxiety and depression*

There was no significant difference in mean scores for trait anxiety, i.e. anxiety proneness, between this sample and normative data obtained from women in the general population (Knight *et al.*, 1983) but patients did score significantly higher on the state measure, i.e. current anxiety, of the STAI ($t=2.95$, $df=70$, $P<0.01$) (see Table 1).

The scores obtained by these women on the psychological distress subscale of the RSCL were comparable to those reported by the authors among female cancer patients receiving chemotherapy (de Haes *et al.*, 1990) but tended to be higher than the mean scores they reported for disease free women ($t=1.70$, $df=88$, $P>0.1$) (see Table I).

Thirty-three per cent of the women scored as depressed on the BDI (i.e. score $>4$) and 13% were moderately – severely depressed (i.e. score $\geqslant 8$).

There were no significant differences by treatment, age or time since diagnosis in any of these measures of mood disturbance.

### *Specific worries about cervical cancer*

The most common worries endorsed by more than 25% of the sample are shown in Table II. The most common concerns were also the most serious. By far the greatest of these was the continuing fear of recurrent disease. No woman was free of worries related to cervical cancer and most had several concerns. Summing scores over 16 items gave a mean score of 11 (s.d. = 8) from a possible total of 48.

**Table I** Anxiety and distress scores: patients and published norms

| | *Mean* | *s.d.* | *n* |
|---|---|---|---|
| Trait anxiety (STAI) | | | |
| Cervical ca sample | 38.2 | 10.7 | 71 |
| Norms (Knight *et al.*, 1983) | 36.9 | 8.9 | 586 |
| State anxiety (STAI) | | | |
| Cervical ca sample | 37.3 | 10.8 | 70 |
| Norms (Knight *et al.*, 1983) | 33.5 | 8.6 | 579 |
| Psychological distress (RSCL) | | | |
| Cervical ca sample | 14.2 | 5.5 | 67 |
| Disease free patients (female)[a] | 12.3 | 4.3 | 23 |
| Chemotherapy patients (female)[a] | 13.8 | 4.0 | 72 |

[a](de Haes *et al.*, 1990)

*Relationship between physical symptoms, psychological distress and functional outcome*

Summed composite scores obtained for physical complaints (RSCL) and for the functional status items were highly correlated. Psychological variables were also significantly correlated both with physical complaints and functional status (Table III).

*Sexual function*

Sixty-one of the sample were sexually active at the time of the assessment. Relative to what they regarded as usual for them in the same relationship prior to the appearance of any symptoms of cervical cancer almost half reported deterioration in their sexual function. All phases of the sexual response cycle were affected and all the differences between premorbid and post treatment ratings were highly significant ($P<0.005$). There was a minority of women (7%) who reported an improvement in their sexual function following treatment.

Although radiotherapy treated patients initially appeared to have more sexual problems, adjusting for premorbid function and age a proportional odds multiple regression analysis demonstrated that the only significant differences between treatment groups were to be found in the experience of pain on intercourse and of enjoyment. Radiotherapy patients were significantly more likely to report pain and loss of sexual pleasure after treatment (Table IV).

The women's ratings of their current difficulties in sexual function were highly correlated both with their total score for physical symptoms on the RSCL and with psychological distress scores (Table V).

The majority of these sexually active women (40/61) still considered their sexual relationship important although 26 of them experienced negative thoughts and emotions about sexual contact and 13 of them were critical of their partner's role in their sexual difficulties. Ten women at the time of the study would have like sexual counselling.

**Table II** Percentage prevalence of worries about cervical cancer among successfully treated patients ($n = 83$)

| *Personal worries* | *%* | *Relationship/sexual worries* | *%* |
|---|---|---|---|
| Disease recurrence | 91 | Sex causing pain | 60 |
| General health | 61 | Sex causing recurrence | 37 |
| Self blame | 39 | Partners attitude | 32 |
| Loss of self confidence | 37 | Blame partner for disease | 29 |
| Feel old | 29 | Less sexually attractive | 29 |
| Less attractive as a person | 28 | | |

**Table III** Correlation of total scores for physical complaints and functional status with psychological distress scores ($r_s$ = Spearman's rho)

| | *Physical symptoms* $r_s$ | *Functional status* $r_s$ |
|---|---|---|
| Physical symptoms | | 0.34[b] |
| BDI depression | 0.63[a] | 0.65[a] |
| STAI trait anxiety | 0.52[a] | 0.40[a] |
| STAI state anxiety | 0.35[b] | 0.39[a] |
| Worries about cervical cancer | 0.48[a] | 0.36[b] |

[a]$P<0.001$; [b]$P<0.02$.

**Table V** Correlation of scores for physical symptoms, anxiety and depression with ratings of current sexual function (Spearman's rho)

| | *Frequency* | *Pain* | *Enjoyment* |
|---|---|---|---|
| Physical symptoms | − 0.46[a] | 0.56[a] | − 0.39[a] |
| Trait anxiety | − 0.37[a] | 0.31[b] | − 0.40[a] |
| State anxiety | − 0.34[a] | NS | − 0.39[a] |
| Depression | − 0.32[b] | 0.27[c] | − 0.37[a] |

[a]$P<0.01$. [b]$P<0.05$. [c]$P=0.06$.

*Partner involvement*

Sixty-three per cent of the 63 women with partners reported that their partner had never attended a clinic visit with them nor spoken with their doctor, although it should also be noted that 41% did not want their partners to attend with them. Women were asked to rate the amount of support they had from their partners as: more than they needed, adequate for their needs, less than they needed or totally lacking. Three aspects of support were assessed: practical help, affection/emotional support and communication. While the men had for the most part offered adequate practical help (90%) and emotional support (72%) 44% felt unable to talk adequately to their partners about their feelings in relation to cervical cancer and 63% reported that their partners were unable to talk to them about these feelings.

*Additional help required*

The majority of the sample (>50%) felt they needed additional information about the cause of cervical cancer and the risk of recurrent disease after treatment. A significant minority felt that they needed help in coming to terms with the diagnosis, in knowing what to do to help themselves recover from treatment and with their sexual relationship. Forty-two per cent had changed their health care practices since completing treatment and 49% would have liked to have had counselling. Given a choice of how counselling might be offered 40% said they would have liked more counselling from staff while 37% would have liked to meet another woman who had previously had the same treatment. Only 18% said they would have been willing to attend a group.

*Feedback*

Eighty brief feedback questionnaires were sent to participants and 54 returned, a response rate of 68%. Of the respondents all but one said they did not mind being asked to participate in the study and would be willing to do so again. The most common (35%) and most popular (69% of all first choices) reason for taking part was to help others. Three out of four respondents found the study interesting and relevant to their own concerns and more than half found it helpful to them to participate. Only three patients had a problem with participation in the study – all three mentioned the anxiety raised by

**Table IV** Change in sexual experience, pre-post-treatment, retrospectively assessed ($n = 61$)

| | *% Ratings* | | *RT* vs | *Adjusted for age* |
|---|---|---|---|---|
| | *Improved* | *Deteriorated* | *Surgery*[a] | *and premorbid function*[b] |
| Interest | 5 | 49 | $P<0.05$ | NS |
| Frequency of intercourse | 2 | 47 | $P<0.05$ | NS |
| Arousal | 11 | 42 | NS | NS |
| Lubrication | 7 | 46 | NS | NS |
| Orgasm | 7 | 49 | NS | NS |
| Pain | 9 | 36 | $P<0.05$ | $P<0.01$ |
| Enjoyment | 7 | 47 | $P<0.01$ | $P<0.01$ |

[a]$P$ values given for Mann Whitney test. [b]$P$ values for proportional odds multiple regression.

receiving unsolicited mail with the hospital post mark. These women also found some questions upsetting. Sixty per cent of respondents, including the three who found some questions upsetting reported feeling better for having talked about their experience.

### Discussion

This retrospective study offers a useful description of the psychosexual outcome for a sample of cured cervical cancer patients but as treatment was not randomly assigned only limited conclusions can be drawn about the relative morbidity of the treatment procedures employed.

Some persistent physical morbidity is to be expected in a proportion of patients undergoing either Wertheim's hysterectomy or pelvic radiotherapy. The 23% of these women who complained of persistent urinary urgency and frequency was higher than in other series (Fiorica *et al.*, 1990) but no significant differences were found in the prevalance of urinary complaints between the treatment modalities. Radiation damage to the bowel resulted in a higher prevalence of complaints of persistent diarrhoea in the RT treated group. The principal physical complaints of tiredness and lack of energy have been reported in other groups of cancer survivors (e.g. Fobair *et al.*, 1986). Although fatigue is recognised as a late radiation effect this is clearly inadequate to explain our data since no significant differences were observed between treatment groups. Women themselves informally mentioned hormonal factors and weight gain in connection with their lack of energy. No age differences were observed but it is relevant that 38% of the sample described sleeping difficulties. Psychological factors were highly correlated with subjectively reported physical complaints.

Although no more anxiety prone than a sample of women drawn from the general population (Knight *et al.*, 1983) the women in this sample showed significantly more state i.e. current anxiety. Their scores on the psychological distress subscale of the RSCL were high compared with other groups of disease free female cancer patients off treatment (de Haes *et al.*, 1990; Watson *et al.*, 1992). The prevalence of depressive disorders in the general population is quoted as 6% (Mermelstein & Lesko, 1993). The 13% of this sample who scored as moderately-severely depressed on the Black Depression Inventory warranted more thorough clinical assessment.

In a retrospective study it is not possible to determine cause and effect. It is not clear whether those with more persistent side effects become more distressed or whether distress increases the subjective reporting of physical complaints. It was interesting that trait anxiety, which as an enduring characteristic may be presumed to antedate the diagnosis of cancer, correlated more highly with physical complaints than did transient state anxiety but clinical experience supports a two-way interaction between soma and psyche. What is important is the combined impact of these factors on the functional outcome for these women.

It was troubling that as many as 60% of this sample of potentially cured women, whose average age was 45 years, had not resumed all their former activities. Six of 51 who formerly had had paid employment had stopped work. A further 11 women reduced their hours or work responsibilities following treatment. It is not clear to what extent this may have represented a welcome opportunity for release for these women from arduous or unrewarding jobs but similar proportions also failed to resume their former social and leisure activities suggesting some persistent impairment of function. Functional status did not vary with time suggesting that where problems exist they are likely to persist. Early assessment of rehabilitation difficulties is indicated.

While some physical morbidity may be an inevitable and acceptable price to pay for curative therapy the question needs to be raised of whether the functional outcome for these women could be improved, for the same physical end state, by relief of some of their psychological distress.

Almost all patients reported persistent anxiety about recurrence of the disease and the majority worried about their health generally. More than one-third carried the additional burden of believing themselves responsible for the onset of their disease. These concerns were reflected in a desire for more information about what is known about the cause of cervical cancer and factors influencing the risk of relapse. Access to accurate information would go some way to relieving these women's anxieties. Forty-two per cent had changed some aspect of their health care behaviour after treatment and a further 27% asked for more information about what they could do to help themselves. These women are highly motivated to accept health education advice covering such issues as diet, exercise, coping with menopausal change or stopping smoking.

The challenge to the self which cervical cancer represented for some women was reflected in reports of loss of self confidence, reduced attractiveness and a sense of accelerated ageing. Similar findings having been reported following hysterectomy for benign disease (Lalinec-Michaud & Engelsmann, 1985; Kincey & McFarlane, 1984) and it has been suggested that women who have diversified interests besides the traditional gender role definition cope better, further underlining the relevance of assessing functional outcome in this patient population.

In common with other studies a deterioration in sexual relationships was found for both surgically and radiotherapy treated patients. Of course there are serious limitations on the conclusions which can be drawn from a retrospective study in which women are asked to rate their subjectively recalled premorbid sexual relationships. Data are presented only for those women who were sexually active and who remained in the same partnership.

What was striking was the number of potentially remediable factors which militated against the reestablishment of a satisfactory sexual relationship. Pain on intercourse was a particular problem for radiotherapy treated patients. Eighty-three per cent had been offered a vaginal dilator and of them 83% reported complying with its use as recommended. Current practice now ensures that all women are instructed in the use of dilators. However psychological as well as physical factors contributed to subjectively reported sexual difficulties.

A substantial proportion ($>\frac{1}{3}$) of the sample worried that resuming their sexual relationship might provoke a recurrence of disease. Given that more than 25% of the women described feeling less sexually attractive, that a similar proportion blame their partners for transmitting the disease and that communication between partners about cervical cancer is commonly experienced as unsatisfactory, it is not surprising that sexual difficulties occur and persist after treatment. Although the small number of women who were clinically depressed would be likely to benefit both emotionally and in their sexual response from psychotropic medication it seems likely that greater numbers could benefit from the opportunity to talk about their sexual concerns.

The women's partners had been practically helpful and willing to give general moral support but were often unable to discuss the women's central concerns. It appeared the men had not directly been offered and had not sought the opportunity to talk with the women's doctors and many women felt their partners had very little information about their disease and treatment. Not surprisingly then communication is compromised, misapprehensions persist and sexual relationships founder.

Concern is often expressed that in routine clinical practice staff lack the time and skill to deal with psychological or sexual concerns although the literature suggests that giving patients the opportunity to express such concerns can be preventative of problems as well as therapeutic (Auchincloss, 1989). Increasing attention is being given to improving training in communication skills to this end (Fallowfield, 1991). This point was underlined by the 60% of respondents in this study who found taking part in a single research interview therapeutic for them.

The indications for offering more specific counselling to women being treated for cervical cancer were recently argued by Corney et al. (1993). Others (Capone et al., 1980; Cain et al., 1986; Schover et al., 1987) have claimed worthwhile results following a variety of brief intervention strategies. A recent survey of cancer counsellors found that under trained and unsupported staff were often overworked to provide ill defined counselling services the effectiveness of which was not evaluated (Fallowfield, 1991). Our sample were not very enthusiastic about group support, perhaps because of the intimate nature of some of their concerns.

We have therefore sought more accurately to define the onset, course and duration of these womens concerns, in a prospective study which is now nearing completion. The results of both studies will inform the evaluation of a planned intervention. It seems likely that at least a proportion of post treatment morbidity can be reduced for these women. The challenge is to find the most cost effective means by which this can be achieved to give the patients the fullest functional benefit of potentially curative therapy.

### References

AUCHINCLOSS, S. (1989). Sexual dysfunction in cancer patients: issues in evaluation and treatment. In *Handbook of Psychooncology*. Holland, J.C. & Rowland, J.H. (eds), pp. 383–413. Oxford University Press: New York.

BECK, A.T. & BEAMESDERFER, A. (1974). Assessement of depression: The Depression Inventory. In *Psychological Measurements in Psychopharmacology. Mod. Probl. Pharmacoypysychiat.* Pichot, P. (ed.) Vol. 7, pp. 151–169. Karger: Basel.

BOS-BRANOLTE, G., ZUEKSTRA, E.M., RIJSHOUWER, Y.M. & DUIVENVOORDEN, H.J. (1988). Psychotherapy in patients cured of gynaecological cancers. In *Supportive Care in Cancer Patients: Recent Results in Cancer Research*. Senn, H.J. & Schmid, L. (eds) Vol. 108, pp. 277–288. Springer Verlag: Berlin.

CAIN, E.N., KOHORN, E.L., QUINLAN, D.M., LATIMER, K. & SCHWARTZ, P.E. (1986). Psychosocial benefits of a Cancer Support Group. *Cancer*, **57**, 183–1889.

CAMPION, M.J., BROWN, J.R., MCCANCE, D.J., ATIA, W., EDWARDS, R., CUZICK, J. & SINGER, A. (1988). Psychosexual trauma of an abnormal cervical smear. *Br. J. Obs. Gynae.*, **95**, 175–181.

CAPONE, M.A., GOOD, R.S., WESTIE, K.S. & JACOBSON, A.F. (1980). Psychosocial rehabilitation of gynaecologic oncology patients. *Arch. Phys. Med. Rehab.*, **61**, 128–132.

CORNEY, R.H., CROWTHER, M.E., EVERETT, H., HOWELL, A. & SHEPHERD, J.H. (1993). Psychosexual dysfunction in women with gynaecological cancer folowing radical pelvic surgery. *Br. J. Obs. Gynae.*, **100**, 73–78.

DE HAES, J.C.J.M., VAN KNIPPENBERG, F.C.E. & NEIJT, J.P. (1990). Measuring psychological and physical distress in cancer patients: structure and application of the Rotterdam Symptom Checklist. *Br. J. Cancer*, **62**, 1034–1038.

FALLOWFIELD, L. (1991). Counselling patients with cancer. In *Counselling and Communication in Health Care*. Davis, H. & Fallowfield, L. (eds), pp. 253–269. J. Wiley & Sons: Chichester.

FIORICA, J.V., ROBERTS, W.S., GREENBERG, H., HOFFMAN, M.S., LA POLLA, J.P. & CAVANAGH, D. (1990). Morbidity and survival patterns in patients after radical hysterectomy and postoperative adjuvant pelvic radiotherapy. *Gynaelogic Oncol.*, **36**, 343–347.

FOBAIR, P., HOPPE, R.T., BLOOM, J., COX, R., VORGHESE, A. & SPIEGAL, D. (1986). Psychosocial problems among survivors of Hodgkin's disease. *J. Clin. Oncol.*, **4**, 805–814.

KINCEY, J. & MCFARLANE, T. (1984). Psychological aspects of hysterecomy. In *Psychology and Gynaecological Problems*. Broome, A. & Wallace, L. (eds), pp. 142–159. Tavistock Publications: London.

KNIGHT, R.G., WAAL MANNING, H.J. & SPEARS, G.F. (1983). Some norms and reliability data for the State-Trait Anxiety Inventory and the Zung Self Rating Depression Scale. *Br. J. Clin. Psychol.*, **22**, 245–249.

LALINEC-MICHAUD, M. & ENGELSMANN, F. (1985). Anxiety fears and depression related to hysterectomy. *J. Psychiat.*, **30**, 44–47.

MAGUIRE, P. (1985). Improving the detection of psychiatric problems in cancer patients. *Soc. Sci. Med.*, **20**, 819–823.

MERMELSTEIN, H.T. & LESKO, L. (1983). Depression in patients with cancer. *Psychoonocology*, **1**, 199–215.

SCHOVER, L.R., EVANS, E.B. & VON ESCHENBACH, A.C. (1987). Sexual rehabilitation in a cancer centre: diagnosis and outcome in 384 consultations. *Arch of Sex Behaviour*, **16**, 445–461.

SCHOVER, L.R., FIFE, M. & GERSHEN, D.M. (1989). Sexual dysfunction and treatment for early stage cervical cancer. *Cancer*, **63**, 204–212.

SIEBEL, M., FREEMAN, M.G. & GRAVES, W.L. (1982). Sexual function after surgical and radiation therapy for cervical carcinoma. *Southern Med. J.*, **75**, 1195–1197.

SPIELBERGER, C.D., GORSUCH, R.L. & LUCHENE, R.E. (1970). *State Trait Anxiety Inventory: Manual.* Consulting Psychologists Press Inc. Palo Alto: California.

WATSON, M., LAW, M., MAGUIRE, G.P., ROBERTSON, B., GREER, S., BLISS, J.M. & IBBOTSON, T. (1992). Further development of a Quality of Life measure for cancer patients: The Rotterdam Symptom Checklist (revised). *Psychooncology*, **1**, 35–44.

WEIJMAR SCHULTZ, W.C.M., VAN DE WIEL, H.B.M. & BOUMA, J. (1991). Psychosexual functioning after treatment for cancer of the cervix: a comparative and longitudinal study. *Int. J. Gynaecol. Cancer*, **1**, 37–46.